# Evaluation of the efficacy of HEMO_2_life®, a marine OXYgen carrier for Organ Preservation (OxyOp2) in renal transplantation: study protocol for a multicenter randomized trial

**DOI:** 10.1186/s13063-023-07302-3

**Published:** 2023-05-01

**Authors:** Yannick Le Meur, Emmanuel Nowak, Benoit Barrou, Antoine Thierry, Lionel Badet, Matthias Buchler, Jean-Philippe Rerolle, Leonard Golbin, Agnès Duveau, Jacques Dantal, Pierre Merville, Nassim Kamar, Leïla Demini, Franck Zal

**Affiliations:** 1 Department of Nephrology, Hôpital de La Cavale Blanche, CHRU de Brest, Brest, France; 2 Centre d’Investigation Clinique INSERM CIC 1412, Hôpital de La Cavale Blanche, CHRU de Brest, Brest, France; 3 Public Agency for Clinical Research and Innovation (DRCI), Brest University Hospital, Brest, France; 4 Department of UrologyNephrology and Transplantation, Assistance Publique-Hôpitaux de Paris AP-HP, Hôpitaux Universitaire de La Pitié Salpétrière-Charles Foix, Paris, France; 5 Department of Nephrology, CHU La Milétrie, Poitiers, France; 6 Department of Urology, Hôpital Edouard Herriot, Hospices Civils de Lyon, Lyon, France; 7 Department of Urology, Hôpital Bretonneau, CHRU de Tours, Tours, France; 8 Department of Nephrology, CHU Dupuytren, Limoges, France; 9 Department of Nephrology, CHU de Rennes, Rennes, France; 10 Department of Nephrology and Urology, CHU d’Angers, Angers, France; 11 Department of Nephrology and Urology, CHU de Nantes, Nantes, France; 12 Department of Nephrology and Urology, Hôpital Pellegrin, Bordeaux, France; 13 Department of Nephrology and Urology, Hôpital Rangueil, CHU de Toulouse, Toulouse, France; 14 HEMARINA, Aéropôle Centre, Morlaix, France

**Keywords:** HEMO_2_life®, Clinical trial, Delayed Graft Function (DGF), Transplantation, Ischemia–Reperfusion Injury (IRI), Kidney IRI

## Abstract

**Background:**

Preventing ischemia‒reperfusion injury (IRI) is a major issue in kidney transplantation, particularly for transplant recipients receiving a kidney from extended criteria donors (ECD). The main consequence of IRI is delayed graft function (DGF). Hypoxia is one of the key factors in IRI, suggesting that the use of an oxygen carrier as an additive to preservation solution may be useful. In the OxyOp trial, we showed that the organs preserved using the oxygen carrier HEMO2life® displayed significantly less DGF. In the OxyOp2 trial, we aim to definitively test and quantify the efficacy of HEMO2life® for organ preservation in a large population of kidney grafts.

**Methods:**

OxyOp2 is a prospective, multicenter, randomized, comparative, single-blinded, parallel-group study versus standard of care in renal transplantation. After the selection of a suitable donor according to the inclusion/exclusion criteria, both kidneys will be used in the study. Depending on the characteristics of the donor, both kidneys will be preserved either in static cold storage (standard donors) or on machine perfusion (for ECD and deceased-after-cardiac-death donors (DCD)). The kidneys resulting from one donor will be randomized: one to the standard-of-care arm (organ preserved in preservation solution routinely used according to the local practice) and the other to the active treatment arm (HEMO2life® on top of routinely used preservation solution). HEMO2life® will be used for ex vivo graft preservation at a dose of 1 g/l preservation solution. The primary outcome is the occurrence of DGF, defined as the need for renal replacement therapy during the first week after transplantation.

**Discussion:**

The use of HEMO2life® in preservation solutions is a novel approach allowing, for the first time, the delivery of oxygen to organs. Improving graft survival by limiting ischemic lesions is a major public-health goal in the field of organ transplantation.

**Trial registration:**

ClinicalTrials.gov, ID: NCT04181710. registered on November 29, 2019.

## Administrative information

Note: the numbers in curly brackets in this protocol refer to SPIRIT checklist item numbers. The order of the items has been modified to group similar items (see http://www.equator-network.org/reporting-guidelines/spirit-2013-statement-defining-standard-protocol-items-for-clinical-trials/).

**Table:** 

Title {1}	**Evaluation of the efficacy of HEMO**_**2**_**life®, a marine OXYgen carrier for Organ Preservation (OxyOp2) in renal transplantation: Study protocol for a multicenter randomized trial**
Trial registration {2a and 2b}	ClinicalTrials.gov, ID: NCT04181710. registered on November 29, 2019
Protocol version {3}	February 22 ^nd^, 2022, Version 3
Funding {4}	This study was supported by a grant from the French Ministry of Health (PHRCN 2018), and was cosponsored by HEMARINA
Author details {5a}	^1^Department of Nephrology, Hôpital de la Cavale Blanche, CHRU de Brest, Brest, France. ^2^Centre d'Investigation Clinique INSERM CIC 1412, Hôpital de la Cavale Blanche, CHRU de Brest, Brest, France, ^3^Public Agency for Clinical Research and Innovation (DRCI), Brest University Hospital, Brest, France, ^4^Department of Urology, Nephrology and Transplantation, Assistance Publique-Hôpitaux de Paris AP-HP, Hôpitaux Universitaire de la Pitié Salpétrière-Charles Foix, Paris, France. ^5^Department of Nephrology, CHU la Milétrie, Poitiers, France. ^6^Department of Urology, Hôpital Edouard Herriot, Hospices Civils de Lyon, Lyon, France. ^7^Department of Urology, Hôpital Bretonneau, CHRU de Tours, Tours, France. ^8^Department of Nephrology, CHU Dupuytren, Limoges, France. ^9^Department of Nephrology, CHU de Rennes, France. ^10^Department of Nephrology and Urology, CHU d’Angers, Angers, France. ^11^Department of Nephrology and Urology, CHU de Nantes, Nantes, France. ^12^Department of Nephrology and Urology, Hôpital Pellegrin, Bordeaux, France. ^13^Department of Nephrology and Urology, Hôpital Rangueil, CHU de Toulouse, Toulouse, France. ^14^HEMARINA, Aéropôle Centre, Morlaix, France
Name and contact information for the trial sponsor {5b}	Brest University Hospital Direction de la Recherche et de l’Innovation Avenue Foch 29,609 BREST CEDEX—FRANCE Phone: + 33.2.98.22.39.79 Fax: + 33.2.98.22.31.83
Role of sponsor {5c}	Monitoring of the collected data and screening forms in each participating centre will be carried out by the sponsor. The funding source has no influence on trial design, trial conduct, data handling, data analysis or writing of the manuscript

## Introduction

### Background and rationale {6a}

Kidney transplantation is the treatment of choice for patients with end-stage renal disease, as it provides long-term benefits in terms of patient survival and quality of life [1]. However, this therapy is now a victim of its own success, with an increasing shortage of organs resulting in long waiting times and increased mortality on the waiting list. In 2019 in France, 3643 kidney transplants were performed for approximately 15 000 patients on the waiting list [2]. This has led to an expanded acceptance of donor organs: donors with risks identified as extended-criteria donors (ECD) or deceased-after-cardiac-death donors (DCD). These donors display a higher incidence of primary nonfunction (PNF) [3] and an increased rate of delayed graft function (DGF) [4]. If the causes of graft loss in the long term are multifactorial, involving factors related to the donor and secondary events in the recipient (infection viral, rejection, drug toxicity, hypertension, metabolic complications), acquired lesions secondary to ischemia‒reperfusion arise [5].

Ischemia–reperfusion is a complex pathophysiological process involving hypoxia and/or reoxygenation, ionic imbalance-induced edema and acidosis, oxidative stress, mitochondrial uncoupling, ATP depletion, coagulation and endothelium activation that is associated with a proinflammatory immune response (reviewed in [6]). I/R is also associated with the activation of Toll-like receptors responsible for the recruitment and activation of immune cells from both the innate and adaptive immune systems, the production of proinflammatory cytokines and chemokines and the activation of the complement pathway (reviewed in [7]). The main consequences of renal ischemia–reperfusion are kidney graft PNF and DGF. It also favors acute rejection [8] and the development of interstitial fibrosis [9], which both have an impact on long-term graft outcome [10]. As these adverse effects (AEs) are most severe in nonoptimal grafts (those obtained from ECD or DCD donors), new approaches are needed to improve the recovery and preservation of kidney grafts [11], particularly those from high-risk donors.

Preservation solutions are based on the principle of hypothermia, which is to maintain organs in a solution at 4 °C to reduce metabolism. A recent meta-analysis suggested that the three most popular solutions (UW, HTK and Celsior) bring comparable risks of DGF [12]. Preservation solutions attempt to reduce the damage associated with ischemia‒reperfusion, but none provide a solution to hypoxia or the reoxygenation responsible for damage, whose deleterious effect on graft survival in the long term is well established. For the last five years, hypothermic machine perfusion (HMP) preservation is increasingly being used as an alternative to static cold storage (SCS) for the preservation of grafts obtained from donors. HMP relies on the recirculation of cold preservation solution through the vasculature of the organ in either a continuous or pulsatile manner. Recent studies have shown that the use of machine perfusion improves the recovery of graft function compared with SCS (rate of DGF from 26.5% to 20.8%) and graft survival [13, 14]. Even if machine perfusion preservation might be beneficial in limiting interstitial fibrosis and tubular atrophy [15], this approach can still be improved by increasing the oxygen supply. However, high concentrations of oxygen may favor the production of oxygen free radicals and promote tissue damage [16]. Several approaches have been investigated to supply oxygen to the organ, such as the two-layer preservation method using perfluorocarbons [17–19], the gaseous oxygenation of preservation solution by retrograde persufflation (effectiveness not determined)[20] and, more recently, a portable device for oxygenated perfusion that has been developed and tested in a pig model of autotransplantation [21]. This study clearly showed a benefit of oxygen in terms of renal function recovery and fibrosis at the 3-month kidney biopsy. Along these lines, the COMPARE trial showed that active oxygenation during preservation using oxygenated hypothermic machine perfusion improves kidney function in humans [22].

The medical additive HEMO_2_life®, an oxygen carrier developed by the HEMARINA French Company, is a natural extracellular hemoglobin isolated from the marine lugworm *Arenicola marina*. This biopolymer of high molecular weight (~ 3,600 kDa) has a high oxygen binding capacity, carrying up to 156 oxygen molecules when saturated (compared to 4 for human adult hemoglobin). It releases oxygen according to a simple gradient and exhibits intrinsic superoxide dismutase-like activity, preventing both the generation of potentially harmful heme-protein-associated free radical species and the release of hemoglobin degradation products [23–25]. Prior to transplantation, the addition of HEMO_2_life® to preservation solutions prevents the progressive decline in the dissolved oxygen concentration available for the graft [26]. The efficacy of HEMO_2_life® has been demonstrated in preclinical studies. In a porcine model of autologous transplantation after 24 h of cold ischemia in several preservation solutions, HEMO_2_life® significantly improved the recovery of renal function. After 3 months, animals that received kidneys treated with HEMO_2_life® exhibited better renal function and less interstitial fibrosis, tubular atrophy and inflammation on kidney biopsy [26, 27]. This protein is not glycosylated; does not induce any immunogenic, allergenic, or mutagenic responses; and is degraded into polypeptide chains and heme [23].

In a previous proof-of-concept study (the OxyOp trial), we showed that HEMO_2_life® used as an additive to the preservation solution was safe for the kidney graft and the transplant recipients and that the organs preserved using HEMO_2_life® displayed significantly less DGF [27, 28]. In this OxyOp2 clinical trial, we aim to definitively test and quantify the efficacy of HEMO_2_life® for organ preservation in a large population of kidney grafts.

### Objectives {7}

The present research focuses on the efficacy of HEMO_2_life® added to preservation solution in kidney transplantation.

The primary objective is:

- To quantify the efficacy of HEMO_2_life® used as an additive to standard organ preservation solution to prevent DGF following renal transplantation.

The secondary objective is as follows:

- To assess and compare graft and patient survival in the two groups (standard of care versus HEMO_2_life®)
- To assess the efficacy of HEMO_2_life® on renal parameters compared with the standard of care.
- To assess efficacy in specific populations with different donor types and preservation method (standard donors, ECD, DCD, SCS, machine perfusion, different perfusion solutions).
- To evaluate the impact of HEMO_2_life® on the degree and progression of interstitial fibrosis before implantation and in 3-month biopsies.
- To assess the safety profile of HEMO_2_life® for the graft and the graft recipient.

### Trial design {8}

**Fig. 1:**
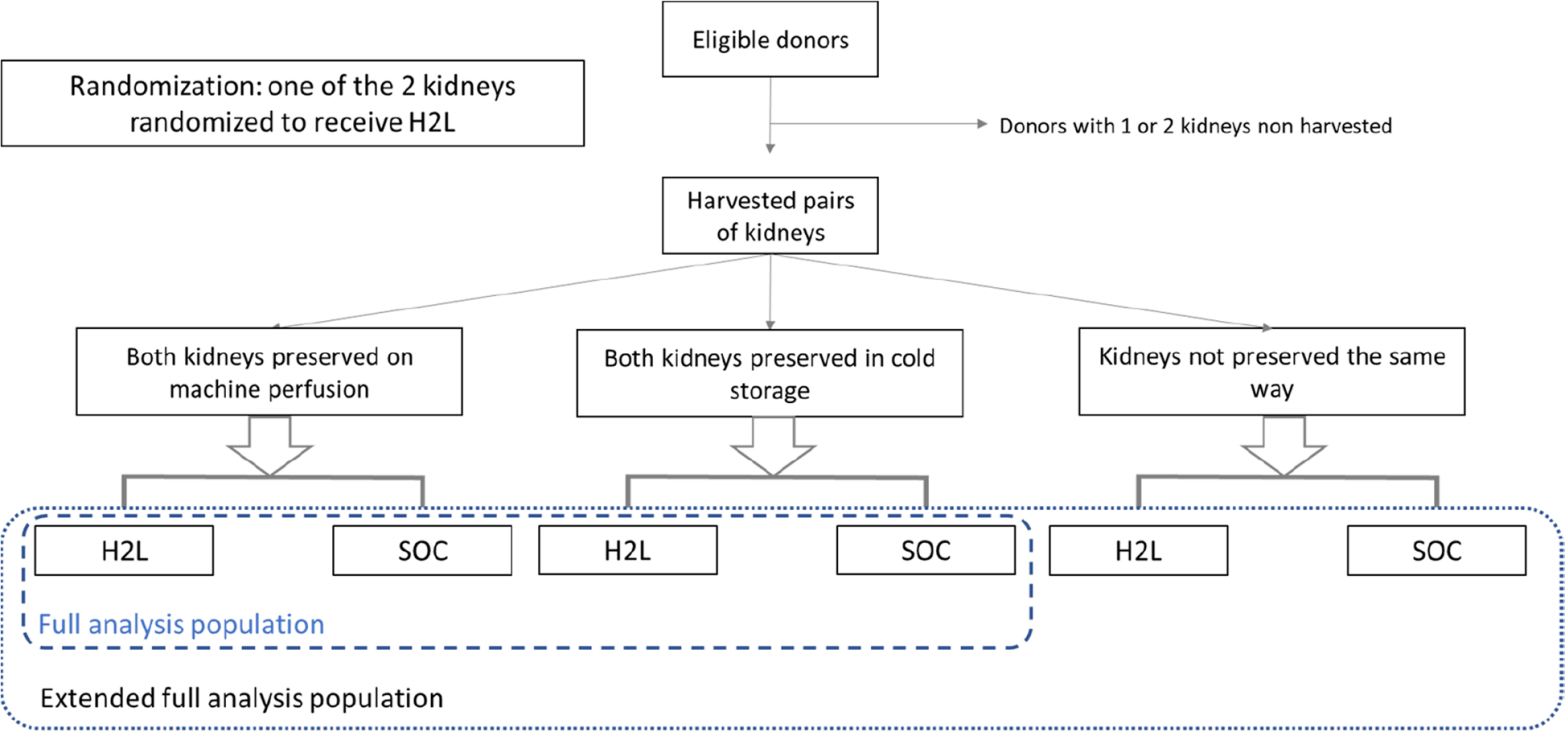
Flowchart of the trial design. H2L: HEMO_2_life®. SOC: Standard of Care

OxyOp2 is a prospective, multicenter, randomized, comparative, single-blinded, parallel-group, superiority study versus standard of care in renal transplantation. After the selection of a suitable donor according to the inclusion/exclusion criteria, both kidneys will be studied. Depending on the characteristics of the donor and following the French recommendations for organ preservation (Agence de la Biomédecine), both kidneys will be preserved either in cold storage or on machine perfusion (Fig. 1). Basically, grafts from standard donors are preserved in cold storage, whereas grafts from extended criteria donors are preserved on machine perfusion. Each kidney will be allocated to a patient following the French organ allocation rules independently of the protocol. In accordance with the rules for assigning kidney transplants in France, one kidney is allocated and transplanted locally (local kidney) in the transplant center where the kidney has been retrieved (local waiting list), whereas the contralateral kidney is allocated based on the national waiting list (national waiting list).

## Methods: Participants, interventions and outcomes

### Study setting {9}

All French centers performing adult renal transplantation will participate in the study. They will be divided into two groups. Thirteen centers (first-line) will be involved in organ procurement, randomization, administration of the study molecule, transplantation of the allocated kidneys and follow-up of the patients, whereas the other 19 centers (second-line) will only transplant the allocated kidneys and follow up the patients. Any graft pair retrieved in the participating first-line centers is eligible for inclusion.

### Eligibility criteria {10}

#### Inclusion criteria

For kidney graft:

- any pair of kidneys retrieved from an adult donor in one of the first-line participating centers.
- any pair of kidneys from a deceased donor after brain or cardiac death (DBD or DCD) who was not opposed of the use of its donation to help scientific research (checking of the French National Registry for Refusal of Organ Donation).

For kidney recipient:

- male or female renal allograft recipient at least 18 years old.
- patient who signed an informed consent form.
- patient receiving one graft from an included pair of kidneys.

#### Exclusion criteria

For kidney graft:

- graft from a living donor.
- graft dedicated to multiorgan transplantation or dual kidney transplantation.
- donor registered in the French National Registry for Refusal of Organ Donation

For patient:

- age less than 18 years.
- refusal to participate in the study.

### Who will take informed consent? {26a}

#### Donor

By definition, it will be impossible to get informed consent from the donors. However, according to French law, the grafts will be included in the study after we check the French National Registry for Refusal of Organ Donation to see if the deceased subject was not opposed to the use of such donation to help scientific research.

#### Recipient

The ethics committee recommended sending a general letter of information about the study to all the patients on the waiting lists of the participating centers. Just before transplantation, the recipients of the graft will be again informed orally by the investigator from the transplant center, and if they accept, they will be asked to sign an informed consent form for the collection of clinical and biological data and conservation of biological samples. A record will be made of which grafts are randomized in the study but grafted to recipients who refuse to give their consent to participate in this research. This file will be fully anonymized and will be limited to collecting information about the grafts, donors, surgical information and incidents for grafts preserved with HEMO_2_life®.

### Additional consent provisions for collection and use of participant data and biological specimens {26b}

All the biological specimens used in the study were collected in the routine follow-up of the patients.

## Interventions

### Explanation for the choice of comparators {6b}

The two kidneys coming from one donor will be randomly allocated: one to the standard-of-care arm (organ preserved in preservation solution routinely used according to the local practice) and the other to the active treatment arm (HEMO_2_life® on top of routinely used preservation solution).

### Intervention description {11a}

HEMO_2_life® will be used for ex vivo graft preservation by diluting it to 1 g/l in preservation solution. For static cold storage, the kidney will be perfused on the back table with 100–150 ml of the preservation solution plus HEMO_2_life® to allow the release of the oxygen carrier inside of the kidney. Then, the kidney will be transferred to the storage container containing the rest of the mix (850–900 ml). For machine-perfused grafts, 1 g of HEMO_2_life® will be added to 1 l machine perfusion solution, and kidney placement on the machine will be performed according to the usual company protocol. In both situations, the kidney will be rinsed just before transplantation with the plain preservation solution (with no HEMO_2_life®) to flush out any residual molecules of HEMO_2_life® and until the liquid becomes clear.

### Criteria for discontinuing or modifying allocated interventions {11b}

Not applicable, treatment is administrated ex vivo.

### Strategies to improve adherence to interventions {11c}

Not applicable, treatment is administrated ex vivo.

### Relevant concomitant care permitted or prohibited during the trial {11d}

The study does not forbid any treatment. No specific immunosuppressive regimen is imposed by the protocol, and the immunosuppressive strategy is left to the discretion of the renal transplant center. All concomitant medications taken during the study will be documented in the patient records and in the eCRF.

### Provisions for post-trial care {30}

There is no anticipated harm and compensation for trial participation.

### Outcomes {12}

- Primary outcome: The primary outcome is the occurrence of DGF, defined as the need for renal replacement therapy during the first week after transplantation.
- Secondary outcomes: Efficacy and safety secondary outcomes will be collected.

*Efficacy*: for comparison of the two groups and specific subgroups of donors (static preservation, machine preservation, type of preservation solution, ECD, DCD and standard donors) and recipients (diabetes, obese, etc.):

- rate of PNF.
- graft and patient survival at one year.
- rate of biopsy-proven acute rejection at one year.
- DGF, assessed with alternative definitions: more than one dialysis session, need for dialysis except for hyperkaliemia or overhydration reason, time to reach a creatinine value of 250 µmol/l, and number of dialysis sessions.
- renal function (creatinine value and estimated glomerular filtration rate (eGFR)) at Days 7, 14, and 30 and months 3 and 12; areas under the concentration curves (AUCs) of creatinine and eGFR from D0 to D30.
- protocol biopsy analysis (preimplantation and at month 3) in a subgroup of 100 patients per group: A) comparison of the chronic scores of tubule-interstitial damage according to the Banff classification [29]: tubular atrophy (ct score ranging from 0 to 3) and chronic interstitial fibrosis (ci score ranging from 0 to 3); and of vascular damage: fibrous intimal thickening (cv score ranging from 0 to 3) and arteriolar hyalinosis (ah score ranging from 0 to 3). B) Analysis of interstitial fibrosis using automated quantitative image analysis (Institut Pasteur-France) and comparison of the progression of interstitial fibrosis in a subgroup of 100 patients per group.
- quality of life: at 1, 3 and 12 months, using the generic self-administered questionnaire EQ-5D and a specific questionnaire for renal transplant recipients in the French language: the ReTransQol (RTQ) [30].

#### Safety

The safety profile of the medical device HEMO_2_life® will be assessed. In particular, all incidents and events of interest occurring during the use of HEMO_2_life® will be collected, including packaging issues, difficulty of mixing HEMO_2_life® into the preservation liquid, perfusion issues, machine perfusion issues, macroscopic aspects of the preservation solution containing HEMO_2_life®, contamination of the conservation solution, macroscopic aspects of the graft before transplantation, and surgical difficulties.

Grafts and patient safety will be investigated by collecting all AEs during the first month and all serious AEs up to one year. These events will be analyzed by the sponsor safety and biovigilance department.

### Participant timeline {13}

The kidney recipient will be followed from transplantation to 1 year after transplantation. The clinical and laboratory data of patients will be collected at D0, D1, D3, D7, D14, M1, M2, M3 and M12 (see Table 1).

**Table 1:**
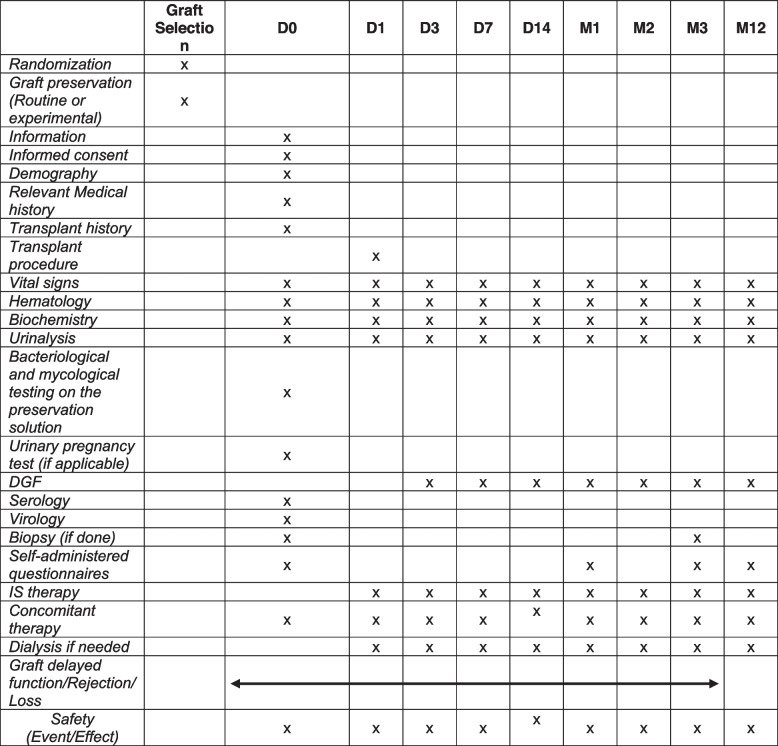
The clinical and laboratory data of patients will be collected at D0, D1, D3, D7, D14, M1, M2, M3 and M12. DGF: Delayed graft function

### Sample size {14}

This sample size is based on the confirmatory testing strategy described below, according to Alosh and Huque’s methodology [31]. This methodology allows us to make inferences on the overall population and/or on a prespecified subgroup (graft preserved in cold storage) while controlling for the familywise error rate (FWER).

This proposed methodology requires first testing global efficacy at a reduced alpha level, chosen here to be 0.03. In the 3 cumulating years 2015 to 2018 the mean rate of DGF in France was 28% according to the data of Agence de la Biomédecine (https://rams-archives2019.agence-biomedecine.fr/sites/default/files/2021-01/LE%20RAPPORT%20M%C3%89DICAL%20ET%20SCIENTIFIQUE%20DU%20PR%C3%89L%C3%88VEMENT%20ET%20DE%20LA%20GREFFE%20EN%20FRANCE2.zip). The sample size calculation rests on an expected DGF rate of 28% in the control arm and a 15% rate in the HEMO_2_life® arm. To guarantee 80% power to detect such a difference at this first step, the minimum sample size per group is 180 evaluable grafts. Expecting 20% of nonevaluable inclusions, a total of 450 patients (225 pairs) are needed.

### Recruitment {15}

We anticipate 10 to 15% of preemptive grafts in the protocol. These kidneys will not be analyzed in the paired analysis for the primary endpoint because they are less likely to require hemodialysis. Therefore, enrollment will be continued until the required sample size in terms of evaluable paired kidneys (180 pairs) is achieved. In total, we expect to include approximately 260 donors in the study.

Nonevaluable inclusions include discordant pairs according to the type of conservation (cold storage/perfusion machine) as well as nontransplanted grafts.

## Assignment of interventions: allocation

### Sequence generation {16a}

Randomized allocation will be achieved through "Ennov Clinical", a secure server with access to the e-CRF, by the retrieving investigational team. The randomization will be stratified according to the donor type (standard donor/ECD/DCD) and the destiny of the kidney (local kidney/contralateral kidney).

### Concealment mechanism {16b}

Use of a validated password website using a secure server will ensure concealment.

### Implementation {16c}

The surgeon in charge of the organ procurement is responsible for the randomization and the administration of the medical device.

## Assignment of interventions: Blinding

### Who will be blinded {17a}

The use of HEMO_2_life® results in a red coloration of the preservation solution, so a double-blind study is not possible. This explains why we designed a single-blind study: the graft recipient will not be informed about the allocated arm.

### Procedure for unblinding if needed {17b}

The surgeon performing the renal transplantation is not blinded so unblinding will not be necessary.

## Data collection and management

### Plans for assessment and collection of outcomes {18a}

The kidney recipient will be followed from transplantation to 1 year after transplantation. All patients will be followed up. For safety reasons, we plan to follow each patient who has received a graft that has been perfused and stored in a preservation solution containing HEMO_2_life®, even if they refuse to participate in the study. In this case, only graft, donor and surgical information and AEs will be collected and recorded in a fully anonymized database, as the patients will not have given their informed consent for biological and clinical data collection.

The following data will be recorded: demographic data, medical history of the donor and the recipient, transplant surgical procedure and complications, vital signs, treatments given, and laboratory tests. Renal function will be assessed by measuring serum creatinine and eGFR using the MDRD formula [32] before surgery; at Days 0, 3, 7, 15, and 30 (but every extra creatinine measurement during the first month will be recorded as well); and at M3 and at M12 (Table 1). PNF, DGF, slow recovery of renal function, acute rejections and transplant dysfunctions will be recorded throughout the study. Biopsy analysis (at least before implantation and at month 3, performed routinely by the centers) will be analyzed by Banff classification [29] and by analyzing lesion evolution, especially that of interstitial fibrosis between the two biopsies (including quantification).

### Plans to promote participant retention and complete follow-up {18b}

For safety reasons, we plan to follow each patient who has received a graft that has been perfused and stored in a preservation solution containing HEMO_2_life®, even if they refuse to participate in the study. In this case, only graft, donor and surgical information and AEs will be collected and recorded in a fully anonymized database, as the patients will not have given their informed consent for biological and clinical data collection.

### Data management {19}

Data will be entered into the web-based electronic Case Report Form (eCRF) (……) under the supervision of the trial site investigators at each participating center. A Clinical Research Assistant (CRA) appointed by the sponsor will be in charge of the study data collection in writing, data recording, data saving and reporting in accordance with the sponsor Standardized Operating Procedures as well as the Good Clinical Practice guidelines and the in force legislation and laws. The investigator and the members of his/her team agree to be available during all the routine and planned Quality Control (QC) visits by the CRA. During these visits, the followings will be audited:

- Signed informed consent
- Compliance of the study’s protocol and its described procedures
- QC of the collected data into the e-CRF: accuracy, missing data, consistency between these data and those of “source” (medical files, original of the laboratory results, etc.)

### Confidentiality {27}

The QC people as well as the investigators are subject to professional confidentiality (according to the French penal code articles 226–13 and 226–14). During the research or at its end, the collected data on the individuals undergoing the trial sent to the sponsor by the investigators (or any other specialized participant) will be anonymized.

### Plans for collection, laboratory evaluation and storage of biological specimens for genetic or molecular analysis in this trial/future use {33}

No specific biological samples were taken for this study. We verified before the start that all the data needed for the analysis were part of the routine blood samples performed in the transplant centers. Additionally, no biological specimens were stored for genetic or molecular analysis.

## Statistical methods

### Statistical methods for primary and secondary outcomes {20a}

Full Analysis Population:

- All complete and concordant (according to the type of conservation: cold storage or perfusion machine) randomized pairs fulfilling the inclusion criteria with no exclusion criterion.
- Preemptive grafts will be excluded
- Randomized pairs who do not receive HEMO_2_life® will be excluded, provided that HEMO_2_life® cancellation is not related to graft function.

Extended Full Analysis Population (exploratory):

- All randomized grafts fulfilling the inclusion criteria and no exclusion criterion will be included.
- Preemptive grafts will be excluded
- Randomized pairs who do not receive HEMO_2_life® will be excluded provided that HEMO_2_life® cancellation is not related to graft function.

#### Safety population

All subjects who receive any study treatment (including control) but excluding subjects who drop out prior to receiving any treatment will be included. Preemptive grafts will be included in the safety population.

Regarding renal function, only available data will be used in a first step that assumes that death and dialysis will be balanced in the two groups. A sensitivity analysis will then be performed using the last observation carried forward (LOCF) for deceased patients and a fixed value (eGFR = 5) for dialysis patients.

#### Primary efficacy analyses

**Fig. 2:**
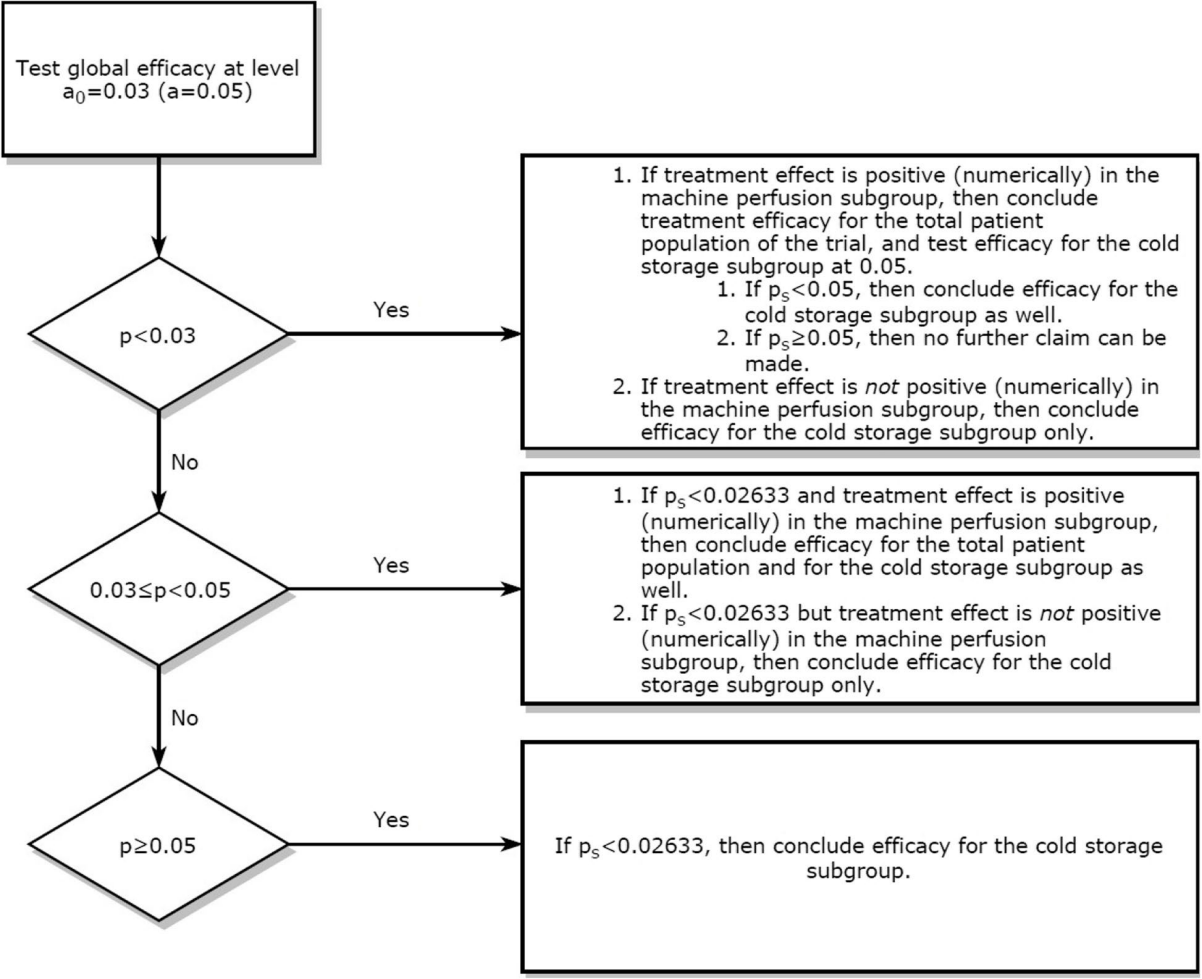
The flowchart depicts the entire confirmatory testing strategy. This implementation will allow the investigator to make inferences on the overall population and/or on a prespecified subgroup (cold storage). The methodology developed by Alosh and Huque [31], which requires meeting some consistency constraints as a prerequisite for testing the next hypothesis, will be used. This proposed methodology requires first testing global efficacy at a reduced alpha level α0. Based on this result and on a consistency constraint within the complementary subgroup (machine perfusion), the efficacy for the cold storage subgroup may also be tested. The variability (standard deviation) is assumed to be similar in the two subgroups. Therefore, the significance level for the subgroup of interest α*S* is derived from the proportions of the subgroups, which are expected to be balanced within the global sample, i.e., 50% cold storage and 50% machine perfusion. This yields an α*S* equal to 0.02633 (two-sided) to control for the familywise error rate (FWER) strongly

As we expect a quantitative interaction regarding the DGF (primary outcome) between the preservation solution (with or without HEMO_2_life®) and the types of preservation (either in cold storage or on machine perfusion), we decided to incorporate this information into the confirmatory testing strategy. This implementation will allow the investigator to make inferences on the overall population and/or on a prespecified subgroup (cold storage). The methodology developed by Alosh and Huque [31] requires first testing the global efficacy at a reduced alpha level α0. Based on this result and on a consistency constraint within the complementary subgroup (machine perfusion), the efficacy for the cold storage subgroup may also be tested. The flowchart below (Fig. 2) depicts the entire confirmatory testing strategy (where p stands for the p value in the total patient population, ps stands for the p value in the prespecified subgroup, being cold storage, and α is the familywise error rate), with the choice of α = 0.03.

The full analysis set (FAS) will be used to perform the primary efficacy analyses. McNemar's test will be used to compare paired proportions (DGF) between patients receiving a kidney preserved with HEMO_2_life® and those receiving a kidney preserved without HEMO_2_life®.

#### Secondary efficacy analyses

DGF proportions will be compared within the machine perfusion subgroup using McNemar's test for paired data in the FAS, as will qualitative secondary outcomes. Quantitative outcomes will be compared between the two groups using the paired t test, or the paired Wilcoxon test whenever the normality condition required for the t test is not satisfied.

Graft survival, death-censored graft survival and patient survival will be described in the 2 groups by Kaplan‒Meier curves. The graft survival analysis treats death as graft failure, whereas the follow-up period is censored at the date of death in the event of death with a functioning graft to calculate death-censored survival. Comparisons will be done using a log-rank test, and a Cox model will allow us to estimate hazard ratios with 95% confidence intervals. A shared frailty model for paired survival data will then be used for exploratory purposes in an attempt to take into account pairing.

The time to reach a creatinine value of 250 μmol/l will be analyzed using Kaplan-Meir curves, log-rank tests and Cox models. Two ways of considering patients with primary nonfunction will be used: excluding them from the analysis and assuming that this value is not reached and censoring at the end of the follow-up period.

For exploratory purposes, two additional approaches will be taken, allowing us to take into account competitive risks (death or graft loss) and pairing: a cause-specific model (usual frailty model for paired survival data) and a regression model of subdistribution hazards for clustered right-censored data [33, 34].

#### Safety analyses

Adverse events will be described using the Preferred Terms (PT) of the MedDRA Hierarchy. The chi-square test or Fisher’s exact test will be used to compare proportions between the two groups.

### Interim analyses {21b}

Not applicable, no interim analysis planned.

### Methods for additional analyses (e.g. subgroup analyses) {20b}

Not applicable, no additional analysis planned.

### Methods in analysis to handle protocol non-adherence and any statistical methods to handle missing data {20c}

#### Missing data

No missing data of the primary outcome are expected except for early (first week) deceased patients. For them, the primary outcome will be imputed as a failure (as a requirement for dialysis during the first week after transplantation).

### Plans to give access to the full protocol, participant level-data and statistical code {31c}

As French law, the datasets generated and/or analyzed during the current study are not publicly available but are available for scientific purpose from the corresponding author on reasonable request after the agreement of the sponsor and the ethics and steering committees.

## Oversight and monitoring

### Composition of the coordinating centre and trial steering committee {5d}

A steering committee has been formed, composed of the principal investigator of each participating center from the first line. It will be responsible for the smooth conduct of the study. It will discuss needed changes to the protocol and will analyze safety reports and alerts prepared by the sponsor or the Independent Data and Safety Monitoring Board composed of three independent experts (IDSMB).

### Composition of the data monitoring committee, its role and reporting structure {21a}

The data will be monitored by an IDSMB of three independent experts in the field of kidney transplantation. The role of the DSMB is to monitor the data from published clinical research that affect patient safety and to ensure that the risk/benefit balance is maintained. Safety reports after the first 25, 75 and 150 pair grafts that have reached the first week after transplantation will be transmitted to the DSMB by the sponsor. This document will be issued within a month by the sponsor safety department and will gather all reported SAEs and AEs, as well as a summary of the incidents/events of interest that occurred during surgeries. A meeting of the IDSMB will be held immediately, and the IDSMB will have to give its recommendation to the steering committee no later than one week after: continuation of study without protocol modification, modification of protocol to improve patient security, and early termination.

### Adverse event reporting and harms {22}

SPIRIT guidance: Plans for collecting, assessing, reporting, and managing solicited and spontaneously reported adverse events and other unintended effects of trial interventions or trial conduct. All adverse events, even unrelated to HEMO2life® occurring during the first month, serious adverse event occurring during the 12 months after transplantation or adverse effect with no time limit will be collected in the study database. When the investigator evaluates the AE as abiding the definition of serious AE, he/she will declare it to the safety Department of the Brest University Hospital. The sponsor must establish once a year during the duration of the clinical trial or on request, an Annual Security Report (ASR) on the clinical trial concerned. He must send it to Agence Nationale de Sécurité du Médicament (ANSM) and Ethic Committee. Its objective is to describe concisely any new information relevant to the safety and assess the safety of persons participating in this Clinical trial.

### Frequency and plans for auditing trial conduct {23}

The trial will be supervised by the sponsor. Research assistants from Brest University Hospital will regularly perform on-site checks of adherence to the protocol and accuracy of the recorded data. Newsletters will be regularly sent to all participants to provide support, information and to recall key instructions. Safety reports after the first 25, 75 and 150 pair grafts will be transmitted to the DSMB by the sponsor. A meeting of the IDSMB will be held immediately, and the IDSMB will have to give its recommendation to the steering committee no later than one week after.

### Plans for communicating important protocol amendments to relevant parties (e.g. trial participants, ethical committees) {25}

The sponsor’s approval is required, in case of any substantial modification applied to the protocol by the investigator. Prior to the trial implementation, the sponsor will be in charge of obtaining the Ethic Committee approval and the regulatory authorization within their respective framework. These modifications will be notified to the participating centers.

### Dissemination plans {31a}

Communications and scientific reports about the study will be elaborated under the principal investigator’s responsibility after co-investigators’ approval. All actors who have contributed substantially in study design, data collection, data analysis and interpretation, manuscript preparation and critical revising, and in final manuscript version approval, will be affiliated as authors. The sponsor and funders will be acknowledged in the published manuscript. Publishing rules will follow the international recommendations (N Engl J Med, 1997; 336(4):309–15).

## Discussion

The use of HEMO_2_life® in preservation solutions is a novel approach, allowing for the first time the delivery of oxygen to organs in storage. Preclinical studies in different animal models have suggested the perfect safety of the molecule and its beneficial effect on ischemia‒reperfusion phenomena [24, 25]. Clinical HEMO_2_life® tolerance in humans was evaluated in our previous safety study [26, 27]. Therefore, the present randomized controlled trial is necessary to assess the efficacy of the product, particularly in specific populations of donors: ECD and DCD. Additionally, it will be of great interest to evaluate the combination of machine perfusion plus HEMO_2_life®. The opportunity is due to this breakthrough oxygen carrier, HEMO_2_life®, and its ability to reduce lesions induced by hypoxia is of major interest. Improving graft survival by limiting these lesions is a major goal of public health in the field of organ transplantation. Its use for the preservation of other organs, particularly for those prone to IRI, such as hearts and lungs, is also expected.

## Trial status

Due to the Covid pandemia the inclusions that were supposed to start in February 2020 were delayed and the recruitment was difficult until end of 2020. The uncertainty regarding the recruitment and the risk of withdrawal of the study explain that we did not submit the manuscript before. Finally, we included the last patient in July 2022. A total of 520 patients were finally included to obtain the 160 pairs needed for analysis. The last one-year visit of the last patient is scheduled in July 2023.

## Data Availability

Any data required to support the protocol can be supplied on request.

## Abbreviations

AE: Adverse Event
CRA: Clinical Research Assistant
CRF: Case Report Form
DBD: Donor after Brain Death
DCD: Deceased Donor
DGF: Delayed Graft Function
DSMB: Data and Safety Monitoring Board
ECD: Extended-Criteria Donor
eCRF: Electronic Case Report Form
eGFR: Estimated Glomerular Filtration Rate
FAS: Full Analysis Set
FWER: Familywise Error Rate
HMP: Hypothermic Machine Perfusion
HTK: Histidine-Tryptophan-Ketoglutarate
IDSMB: Independent Data and Safety Monitoring Board
IRI: Ischemia‒Reperfusion Injury
PNF: Primary Non Function
QC: Quality Control
SCS: Static Cold Storage
UW: University of Wisconsin
